# Bridging the gap between systems biology and medicine

**DOI:** 10.1186/gm88

**Published:** 2009-09-29

**Authors:** Gilles Clermont, Charles Auffray, Yves Moreau, David M Rocke, Daniel Dalevi, Devdatt Dubhashi, Dana R Marshall, Peter Raasch, Frank Dehne, Paolo Provero, Jesper Tegner, Bruce J Aronow, Michael A Langston, Mikael Benson

**Affiliations:** 1 Department of Critical Care Medicine and CRISMA laboratory, University of Pittsburgh School of Medicine, Scaife 602, 3550 Terrace, Pittsburgh, PA 15261, USA; 2 Functional Genomics and Systems Biology for Health, CNRS Institute of Biological Sciences, 7, rue Guy Moquet, BP8 94801 Villejuif Cedex, France; 3 K.U. Leuven, ESAT/SCD, Kasteelpark Arenberg 10, B-3001 Leuven-Heverlee, Belgium; 4 Department of Public Health Sciences, University of California, Davis, One Shields Ave, Davis, CA 95616, USA; 5 Department of Computer Science and Engineering. Chalmers and Göteborg University, SE 41296, Göteborg, Sweden; 6 Department of Surgery, Meharry Medical College, 1005 Dr. D.B. Todd Boulevard, Nashville, TN 37208, USA; 7 Systems Biology and Bioinformatics Group, University of Rostock, Universitätsplatz 1, 18055 Rostock, Germany; 8 School of Computer Science, Carleton University, 1125 Colonel By Drive, Ottawa, Ontario K1S 5B6, Canada; 9 Computational Biology Unit Molecular Biotechnology Center, University of Torino, Via Nizza 52, I, 10126 Torino, Italy; 10 Institutionen för Medicin, Karolinska Universitetssjukhuset, Solna, 171 76 Stockholm, Sweden; 11 Computational Medicine Center, University of Cincinnati, 3333 Burnet Avenue, Cincinnati, OH 45229, USA; 12 Department of Electrical Engineering and Computer Science, College of Engineering, University of Tennessee, 1122 Volunteer Boulevard, Knoxville, TN 37996, USA; 13 The Unit for Clinical Systems Biology, The Queen Silvia Children's Hospital, Gothenburg 40530, Sweden

## Abstract

Systems biology has matured considerably as a discipline over the last decade, yet some of the key challenges separating current research efforts in systems biology and clinically useful results are only now becoming apparent. As these gaps are better defined, the new discipline of systems medicine is emerging as a translational extension of systems biology. How is systems medicine defined? What are relevant ontologies for systems medicine? What are the key theoretic and methodologic challenges facing computational disease modeling? How are inaccurate and incomplete data, and uncertain biologic knowledge best synthesized in useful computational models? Does network analysis provide clinically useful insight? We discuss the outstanding difficulties in translating a rapidly growing body of data into knowledge usable at the bedside. Although core-specific challenges are best met by specialized groups, it appears fundamental that such efforts should be guided by a roadmap for systems medicine drafted by a coalition of scientists from the clinical, experimental, computational, and theoretic domains.

## Correspondence

Recent years have seen the rise of systems biology as a legitimate discipline. Although consensus exists about what the fundamental tools are (high-throughput data from several biologic scales, high-definition imaging, and computational modeling), no such consensus exists as to what defines the broad agenda of systems biology. A growing awareness is found that, despite such major technologic advances, fundamental obstacles separate systems biology from clinical applications. Bridging these gaps will require a focused and concerted effort. What defines systems medicine as a discipline? What should it seek to accomplish? How should knowledge from disparate sources be assembled into ontologies relevant to systems medicine? How are multiscale data to be synthesized by corresponding multiscale models? What is the burden of proof that such models are valid and predictive of clinically relevant outcomes? Is network analysis a useful tool for systems medicine?

Physicians, basic scientists, mathematicians, statisticians and computer scientists met at the Third Bertinoro Systems Biology workshop [1], sponsored by the University of Bologna, focused on the theme 'Systems Biology Meets the Clinic' to address these questions. Participants sought to identify key challenges facing the successful translation of systems biology to the clinical arena and discussed and debated a roadmap seeking to address them. The meeting, held over a 4-day period, comprised plenary lectures followed by extensive thematic discussions, formal and informal, centered on the theme of systems medicine as a distinct translational discipline [2].

## Defining systems medicine

Workshop participants proposed that systems medicine be defined as the application of systems biology to the prevention of, understanding and modulation of, and recovery from developmental disorders and pathologic processes in human health. Although no clear boundary exists between systems biology and systems medicine, it could be stated that systems biology is aimed at a fundamental understanding of biologic processes and ultimately at an exhaustive modeling of biologic networks, whereas systems medicine emphasizes that the essential purpose and relevance of models is translational, aimed at diagnostic, predictive, and therapeutic applications. Accordingly, advances in systems medicine must be assessed on both a medical and more basic biologic scale, as the correspondence between medicine and biology is intricate. Some seemingly straightforward biologic models may have an important medical impact, although some impressively complex molecular models may not be immediately medically relevant. Whereas systems biology may have so far focused primarily on the molecular scale, systems medicine must directly incorporate mesoscale clinical information into its models; in particular, classic clinical variables, biomarkers, and medical imaging data. As an example, it has become increasingly clear that prognostic and predictive models for malignant tumors using expression data cannot ignore information from classic prognostic indices [3].

Furthermore, because of the necessary multiscale nature of the models bridging embedded levels of organization from molecules, organelles, cells, tissues, organs, and all the way to individuals, environmental factors, populations, and ecosystems, systems medicine aims to discover and select the key factors at each level and integrate them into models of translational relevance, which include measurable readouts and clinical predictions. Such an approach is expected to be most valuable when the execution of all experiments necessary to validate sufficiently detailed models is limited by time, expenses (*e.g*., in animal models), or basic ethical considerations (*e.g*., human experimentation). Systems medicine as a discipline did not emerge from clinical medicine, but draws its relevance from it. Conversely, advances in systems biology created the necessary conditions and tools for the emergence of systems medicine.

Accordingly, although it may be appropriate to position systems medicine as an extension of systems biology from a historical perspective, the former also draws from several other disciplines, such as clinical medicine and population epidemiology, less familiar to systems biologists.

## Scale-specific modeling versus multiscale modeling

Computational models have for the most part attempted to assimilate massive data streams collected by using global measurement technologies (techniques that look at the complete set of genes, transcripts, proteins, metabolites, or other features in an organism) by using high-throughput techniques and have been, by and large, scale specific. Such attempts target the development of predictive mathematic and computational models of functional and regulatory biologic networks. Specific biologic hypotheses can thus be tested by designing a series of relevant perturbation experiments [4]. Clear merit inheres in such an incremental approach, yet its true potential is likely to be realized only when such data-driven, bottom-up approaches are combined with top-down, model-driven approaches to generate new medically relevant knowledge.

An open question is whether integrative systems-biology approaches can reveal underlying principles related to the aforementioned biologic functions. It is probably improper to speak of the existence of biologic laws in the sense of physical laws, yet probably deeper dynamic principles guide the evolution of biologic systems. Energetic and physical constraints play an important role in all scale-specific models. Additional principles at play across multiple scales in biologic systems are far less apparent. Thus, it appears prudent at this stage that top-down and multiscale models seek to recapitulate scale-specific observables. As mentioned previously, if computational models are to be validated by experiments such as randomized clinical trials and become predictive of therapeutic interventions, relevant system observables must be included.

## Ontologies relevant to systems medicine

Considerable attention should be paid to the development of ontologies relevant to systems medicine. Such ontologies must reflect knowledge based on biologic function, rather than on biologic structure. Indeed, structure is permissive to function, and clearly, a wide variety of structures could have evolved, under genetic, molecular, or physical constraints to accomplish a given function. Examples include energy generation and storage and transmission of information.

The recent emphasis on mapping structure into function is vital to the advancement of systems medicine. In addition, it appears that the development of appropriate ontologies could promote a (re)interpretation of empiric evidence in light of such ontologies. As an example, experimental data often appear to support contradictory hypotheses of limited scope, when in fact the evidence can be reconciled under a broader synthesis of the evidence.

Progress in developing meaningful ontologies for systems medicine will challenge our current intuition of the nature of a biologic function. Recent efforts at data reduction for longitudinal expression data, by using principal-component analysis to identify and monitor health and disease "trajectories", represent an attempt at understanding such "eigenprocesses" from a data-driven perspective [5,6]. Typically and unfortunately, such processes have limited intuitive meaning when interpreted through the prisms of currently existing ontologies. Alternatively, existing community (for example, Gene Ontology (GO)) or commercial efforts aimed at developing a phenotype-driven ontology (*e.g*., annotating genes to *a priori *defined functions such as "cell-cycle" or "inflammatory response") are commendable and clearly of great value, although it is apparent that extensive cross-contamination exists between such functional assignments and the response to even the simplest experimental perturbation of functions. Knowledge representations relevant to systems medicine will probably lie within this spectrum, and computational efforts will likely be crucial to their development.

Both data-driven techniques and simulation-based techniques open possibilities of reinterpreting what is meant by biologic function, yielding new knowledge representations. Multiscale models that include phenotypes as inputs or readouts will provide mechanistic insight into the dynamic interplay of such redefined functions, and plausibly suggest phenotypically based therapeutic targets.

## New knowledge and false discovery

Experimental design and statistical analysis should be dealt with rigorously, as they play essential roles in discovery and validation in systems biology and medicine [7]. Study design is often the weakest point of complex molecular studies in systems biology and medicine. For example, patients with a disease such as ovarian cancer may be compared with normal controls to discern aberrant regulation of pathways. If controls are not carefully selected to be comparable with patients demographically and in other covariates (age, sex, income, social class), then differences observed may be attributable to factors other than the disease.

Researchers are often unduly optimistic about sample sizes required to show differences, and they fail to consider many confounding effects. Interindividual variability in humans can be large, often the largest effect in a study. This provides an avenue for exploration of individual effects, leading to personalized medicine, but also can make detection of differences across subjects quite difficult.

High-throughput technologies have introduced new challenges to experimental design and interpretation of results. Avoiding false positives may result in difficulties in identifying true positive. Standard approaches to correcting for multiple-testing on datasets generated by global analysis, such as expression microarray, rely on the incorrect assumption that each value is independent of other values. More recent approaches do not fully resolve this problem [8]. Greatly increasing sample sizes is generally impractical. A more practical approach is to make increased use of *a priori *biologic knowledge, either by trimming the list of analytes to a relatively small number for which the multiple-testing correction is modest, or by testing pathways or groups of genes [9]. This is usually done not by testing every group of genes defined by a GO term or a Kyoto Encyclopedia of Genes and Genomes (KEGG) pathway, but by selectively testing those thought to be of importance. Because this more-focused approach, in its effort to improve specificity, is ontology dependent, it may bear a subjective element as to the certainty of prior knowledge. It, therefore, also carries the risk of lacking sensitivity.

Addressing the previously mentioned challenges may have direct clinical implications. A frequent problem encountered by clinicians is that patients appearing to have the same disease may not respond to the same treatment. Some patients even experience severe adverse effects from the treatment. Variable treatment response is also one of the most important causes of the huge costs involved in drug development. Taken together, these cause both increased suffering and costs. Ideally, physicians should be able, routinely and noninvasively, to measure a few diagnostic biomarkers to personalize medication for each patient. At present, not enough knowledge exists about the causes for variable treatment responses in most common diseases. However, recent studies of genetic markers for response to treatment with anticoagulants indicate that personalized dosage may become a clinical reality within the next 5 to 10 years [10]. The main problems involved in finding markers for personalized dosage are that each complex disease may involve altered interactions between hundreds or thousands of genes that can differ among patients. This heterogeneity may, in turn, depend on both genetic and environmental factors. In addition to this complexity, significant problems are involved in clinical research. Ideally, a study aiming to find markers for personalized medication would involve a known external cause, a key cell type, and a read-out, all of which can be studied experimentally in patient samples.

For most complex diseases, all of these factors are not readily available. It is therefore important to find model diseases, in which all those factors can be studied together in patient samples by using high-throughput technologies and systems biologic principles [11]. Such model diseases might be used to develop and apply the methods required to find markers for personalized medicine.

It also has been suggested that the same methods might be applied to find markers to predict the risk of developing disease [12]. If successful, this may lead to a new era of preventive medicine. Finally, the methods may be of great value for drug development. If it were possible to predict which patients respond to medication, this would result in increased efficacy and reduced risk of not being able to market drugs that have been developed at great cost. Conversely, delineation of patients that do not respond to a medication may help to develop new drugs for that specific subgroup. We suggest that acute inflammatory diseases, such as severe trauma, sepsis, and pancreatitis, might be very attractive test beds for the development of such methods. Similarly, chronic ailments, such as diabetes and other autoimmune disorders, meet several of the criteria mentioned earlier and are of prominent clinical and societal relevance.

## Network analysis

A network represents a set of objects and their mutual relations. Much biologic and medical knowledge can be naturally represented as networks: protein-interaction networks, metabolic networks, gene co-expression networks, disease networks, and many more. Growing concerns regard current trends in network analysis in systems biology and potential extension to the clinical arena through the construction of "diseasomes" [13]. Do network representations actually convey new knowledge, or are they just a convenient and eye-catching way to represent data? How can such networks be used to extract new information that is relevant to understanding biologic systems and guiding clinical practice? Are current approaches adequately representing the types of entities and the specific nature of their relations that determine disease pathophysiologic processes? What challenges might be resolved and opportunities opened for both basic research and clinical practice if standards could be broadly adopted in our knowledge representation, data collection, publication, and reasoning, and if fundamental chemical, physical, and biologic entities and processes could be included in network representations? How might this be enabled by the adoption of disease-oriented ontologies? From a mathematic and computational perspective, what topologic, dynamic, and conditional properties could allow the identification of the nodes in a network whose perturbation would yield adversely affected or clinically improved biologic states?

Although the methods used to analyze networks might still be primitive, they are already providing useful information, especially on the genetics of disease. It is now possible to integrate information from various biologic networks to identify genes involved in both mendelian and complex diseases. In such research efforts, careful thought must be given to how network inferences from microarray and other types of data are evaluated. The development of such tools should ideally involve an open dialogue between experimentalists, modelers, and clinicians, who should be able to assess tools best suited to their application. A need exists for systematic benchmark testing and comparative evaluation of the major tools available. For example, current methods tend to focus more on testing performance capabilities over simulated data or for functional enrichment in GO categories that may not be very relevant to clinically relevant phenomena.

The identification of both disease-causative genes and potential therapeutics has begun to be approached by using integrative network-relevant methods for knowledge representation and reasoning [14,15]. Another possibility is the identification of specific interactions that have been extensively validated, a so-called 'gold standard' for the identification of causal, mechanistic, and deterministic factors in a complex network. Some of these issues have been raised within the Dialogue on Reverse Engineering Assessment and Methods (DREAM) initiative [16]. For example, representing gene interactions with graph algorithms may be a useful method to discover parts of a network that are not fully resolved [17]. The biologic plausibility of such representations could then be integrated with other technologies and discussed with basic biologists and clinicians. Another approach is to extend network analysis to evaluate disease-specific ontologies [18].

## Conclusions and recommendations

**Figure 1:**
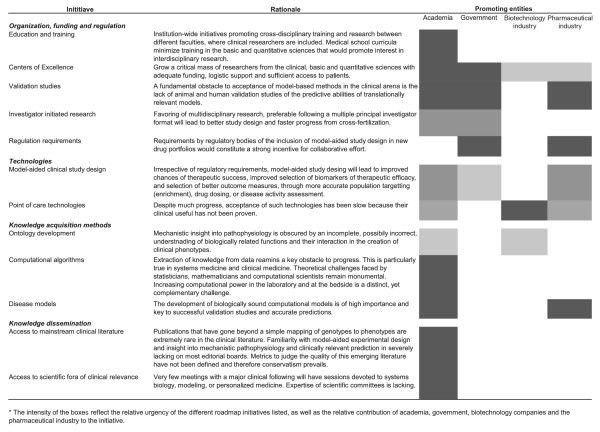
A roadmap for systems medicine.

We consider that improvements in academic infrastructure are sorely needed to facilitate cross-disciplinary translational studies that can someday connect what can be learned by using model organisms with real-time samples from patients. Such improvements include, but are not limited to sufficient funding, appropriate development of mechanisms allowing academic recognition of all participants of transdisciplinary teams, the creation of centers of excellence in systems medicine and specific training programs, and enhancement of the attractiveness of a medical career for individuals with training in quantitative fields. Recognition of systems medicine in the clinical arena should be promoted at the professional society and journal editorial levels. Indeed, whereas bioinformatics exercises can access mainstream clinical literature on account of the value of a significance test, the burden of proof appears disproportionately higher for computational disease and therapeutic models of clinical relevance. Additionally, the construction of a roadmap for systems medicine, facilitated by enhanced visibility in the more clinically oriented medical literature, will be essential to chart effort and progress. We present essential elements of such a roadmap, as well as underlying rationale (Figure 1).

A serious and useful dialogue between the clinic and systems biology has begun. We hope that future developments will provide continuing evidence that the systems-biology community has taken this development to its heart, building systems medicine on a millennium of scholarship and medical tradition.

## Abbreviations

DREAM: dialogue on reverse engineering assessment and methods; GO: gene ontolology; KEGG: Kyoto Encyclopedia of Genes and Genomes.

## Competing interests

The authors declare that they have no competing interests.

## Authors' contributions

All authors contributed text on their specific domains of expertise. GC collated text. All authors reviewed the assembled text for accuracy.
